# Declining freshwater mussel diversity in the middle and lower reaches of the Xin River Basin: Threat and conservation

**DOI:** 10.1002/ece3.5849

**Published:** 2019-11-21

**Authors:** Weiwei Sun, Xiongjun Liu, Ruiwen Wu, Weikai Wang, Yanli Wu, Shan Ouyang, Xiaoping Wu

**Affiliations:** ^1^ School of Life Sciences Nanchang University Nanchang China; ^2^ Key Laboratory of Poyang Lake Environment and Resource Utilization School of Resource, Environmental and Chemical Engineering Ministry of Education Nanchang University Nanchang China; ^3^ School of Resources Environmental & Chemical Engineering Nanchang University Nanchang China

**Keywords:** biodiversity, conservation, freshwater mussels, quantitative, Xin River

## Abstract

Freshwater mussels provide important functions and services for aquatic ecosystems, but populations of many species have been extirpated. Information on biodiversity plays an important role in the conservation and management of freshwater mussels. The Xin River Basin is a biodiversity hotspot for freshwater mussels in China, with more than 43 species known, but populations of which are decreasing. Here, we quantify the diversity of freshwater mussels in the middle and lower reaches of the Xin River Basin and study the correlation of habitat characteristics and freshwater mussel diversity. Compared to the historical period, the number of species, density, and biomass of freshwater mussels decreased 33%, 83%, and 82% in the current period, respectively. Fifty two percent of recorded species were empty shells, and 14 native freshwater mussels were not found in the study area. Four species are currently listed as vulnerable species using IUCN criteria and their global status. The assemblage structure of freshwater mussels exhibits significant spatial differences, and there was a correlation with substrate and physicochemical parameters. The main tributary of the Xin River with higher freshwater mussel diversity should be established as one large protected area because the nestedness component was the main pattern of beta diversity. These results indicated freshwater mussel diversity was declining rapidly, which can help focus conservation effort for freshwater mussel biodiversity.

## Graphical abstract

Freshwater mussels provide important aquatic ecosystem functions and services, but many of their populations are in decline. Here, we systematically quantitative analyze freshwater mussel diversity in the Xinjiang River and explore the correlation of habitat characteristics and freshwater mussel diversity. The results showed that the diversity of freshwater mussels decreased rapidly in the Xinjiang River. Assemblage structure of freshwater mussels exhibits significant spatial differences. The assemblage composition of freshwater mussels was correlated with turbidity, PM, and water temperature. The MT should be established one large protected area because the nestedness component was the main pattern of beta diversity. This study will provide important information for the conservation and management of freshwater mussel biodiversity.

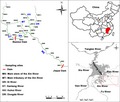

## 1 INTRODUCTION

Human activities, such as habitat fragmentation and loss, water pollution, and overexploitation, have driven global biodiversity decline (Tilman et al., 2017), likely to worsen (Tittensor et al., 2014). These factors in particular were degrading important freshwater ecosystem services and diversity (Dudgeon et al., 2006), but little is known on the status of freshwater invertebrates (Abell et al., 2011; Dudgeon et al., 2006; Sala et al., 2000).

Freshwater mussels (Bivalvia: Unionidae) are a very important part of biodiversity, playing major roles in freshwater ecosystems (Vaughn, 2018). At the same time, they are indicator organism for detecting environmental health (Vaughn, 2012). However, freshwater mussels have been in decline globally in recent decades due to a myriad of human activities (Haag & Williams, 2014; Vaughn, 2018). Two hundred and forty‐seven freshwater mussels have been listed as extinct, endangered, threatened, or near threatened by the IUCN Red List of Threatened Species (IUCN, 2019). In addition, an assessment of the conservation status of freshwater mussels has not been completed in East and South‐East Asia, where 228 species are not under international legal protection (Cao et al., 2018; Zieritz et al., 2018). They are therefore considered to be one of the most threatened freshwater organisms globally (Bogan, 2008).

The Yangtze River Basin is a biodiversity hotspot for freshwater mussels, with more than 80% of them in this area are considered threatened or vulnerable (Liu et al., 2017; Shu, Wang, Pan, Liu, & Wang, 2009; Zieritz et al., 2018). Poyang Lake is the largest river‐connected lake in the Yangtze River Basin with approximately 75% of the endemic freshwater mussel species in China (Li et al., 2019; Sun et al., 2018; Xiong, Ouyang, & Wu, 2013). The Xin River Basin is one of the largest rivers in Jiangxi Province, with the estimated number of freshwater mussel species in the river and supporting citations, flowing into Poyang Lake, which plays an important role in maintaining and supplementing freshwater mussel diversity for the Yangtze River and Poyang Lake (Jin, Nie, Li, Chen, & Zhou, 2012; Li et al., 2019). However, due to disturbances from anthropogenic habitats, which include habitat loss and fragmentation, water pollution, sand dredging, and channelization, populations of many species have been extirpated or greatly reduced in these areas (Liu et al., 2017; Sun et al., 2018). In addition, changes in water levels in river and lake levels coinciding with wet and dry seasons have become more extreme due to climate change. This has affected the assemblage structure of freshwater mussels (Liu et al., 2017; Xiong et al., 2013).

Knowledge on biodiversity plays an important role in the conservation and management of freshwater mussels (Liu et al., 2017; Zieritz et al., 2018, 2016). For example, beta diversity is a key concept for understanding the functioning of ecosystems, the conservation of biodiversity, and the management of ecosystems (Bergamin et al., 2017; Wiersma & Urban, 2005; Xu et al., 2019). Although many studies have qualitatively investigated freshwater mussels in the Xin River Basin (Liu, Ouyang, & Wu, 2008; Tchang & Li, 1965; Wu, Ouyang, & Hu, 1994), there has been little quantitative work on freshwater mussel diversity and their conservation status. Moreover, the correlation between diversity and habitat characteristics has not yet been explored in the Xin River Basin. Here, freshwater mussel abundance and diversity, and correlations with habitat characteristics, were quantified in middle and lower reaches of the Xin River Basin as a basis for conservation and management of the threatened mussel fauna in this Eastern Asian freshwater biodiversity hotspot.

## 2 MATERIALS AND METHODS

### 2.1 Study area

**Figure 1:**
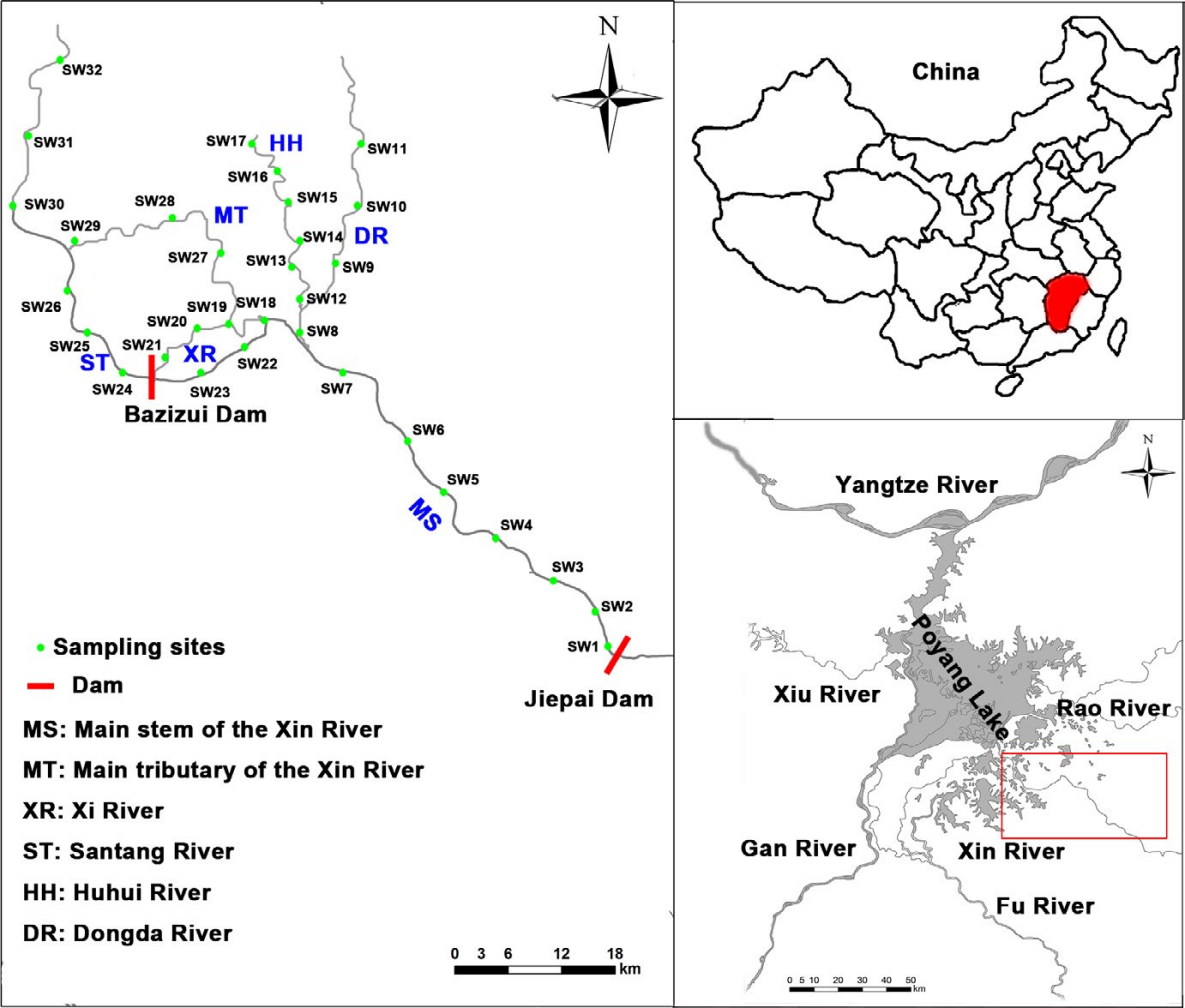
Map showing the study area of the middle and lower reaches of the Xin River Basin, geographic location in China, and in relation to the Yangtze River and Poyang Lake. River codes are the same as in Table 2

The Xin River (116°13′–118°29′E, 27°48′–28°32 N) is one of the largest rivers in Jiangxi Province, China. It has a main channel of 313 km, with a total drainage area of 17,600 km^2^. It has an average precipitation of 1,850 mm/year and is in the middle‐subtropical humid monsoon climate zone. The upper reach of the river is above Shangrao city (115 km), with many streams and coarse gravel on the substrates. The middle reach is from Shangrao city to Yintang city (144 km) with a smooth water flow and stone, sand, and gravel on the substrates. Finally, the lower reach is from Yintang city to Kangshan town (69 km), with a smooth water flow and mud, sand, and gravel on the substrates. Many dams have been constructed in the Xin River, such as Jiepai Dam, Xinzhou Dam, Hongqi Dam, and Jiuniutang Dam currently, and Bazizui Dam and Shuanggang Dam in the future (Figure 1).

### 2.2 Study sites

Study sites were selected in the Xin River Basin considering habitat variation and anthropogenic activities. Six sections (32 study sites) were established in the study area referenced Bureau of hydrology in Jiangxi Province 2007 (Figure 1), and each section included (a) the main stem of the Xin River (section code MS; SW1–SW7), where anthropogenic activities included sand mining, water pollution, and dam construction, and the substrates were sandstone, sand, and gravel; (b) the main tributary of the Xin River (section code MT; SW18–SW19, SW27–SW32), where anthropogenic activities included sand mining, overfishing, water pollution, and dam construction, and the substrates were sandstone, mud, sand, pebble, and gravel; (c) the Xi River (section code XR; SW20–SW21), which had few anthropogenic activities, and the substrates were mud, sand, and pebbles; (d) the Santang River (section code ST; SW22‐SW26), which had many anthropogenic activities including water pollution, agriculture, and urban use, and the substrates were mud and sand; (e) the Huhui River (section code HH; SW12‐SW17), which had many anthropogenic activities including water pollution and urban, and the substrates were mud and sand; and (f) the Dongda River (section code DR; SW8‐SW11), which had anthropogenic activities including water pollution and urban, and the substrates were sandstone, rock, and gravel.

### 2.3 Study methods

Historical data (1965–2013) were collected on the presence–absence of freshwater mussel species in the Xin River Basin from the literature (Tchang & Li, 1965; Wu et al., 1994; Xiong et al., 2013; Xu, 2013; Zhang et al., 2013; Table S1). Historical sampling methods mainly qualitatively collected mussels by searching through the bottom by hand at the shore or in shallow waters (Zhang et al., 2013). Freshwater mussel samples were collected in the current period from October to November 2018. We first asked local people about the presence of freshwater mussels based on pictures or on shells of freshwater mussels historically recorded (1965–2013) from the Xin River. In addition, three repeated samples of freshwater mussels were collected using homemade mussel rakes (60 cm wide, 20 mm mesh, rake tooth spacing 15 mm). The hand‐held mussel rake was thrown into the water and dragged slowly to the shore with uniform speed in the river shallows (0.5–2.0 m of water depth) by the same person to reach approximately the same distance. Then, the towing line distance was measured (the sampling area [15 m^2^] was obtained by multiplying the mussel rake mouth width by the towing line distance [25 m]). Samples were poured into white porcelain dishes for sorting, and specimens were placed into labeled plastic bags. Simultaneously, supplementary qualitative freshwater mussel samples were found visually or by searching through the bottom by hand at the shore or in shallow waters for a minimum of 30 and a maximum of 240 min, covering approximately 200 m of the area sampled close to the river bank. Qualitatively collected mussels were used as the basis for the description of species composition and distribution but were not included in the quantitative analysis. Specimens were identified to the lowest possible taxonomic level (usually species or genus), counted, and weighed with an electronic balance (HANGPING FA1204B; precision: 0.1 g). Freshwater mussel taxonomic levels were mainly based on Liu, Zhang, and Wang (1979), He and Zhuang (2013) and Lopes‐Lima et al. (2018).

### 2.4 Habitat characteristics

Three physicochemical parameters were measured to analyze microenvironmental changes in the study section from October to November 2018. We measured the dissolved oxygen (DO, mg/L), hydrogen ion concentration (pH), turbidity (TURB, NTU+), water temperature (*T*, °C), salinity (Sal, mg/L), and electrical conductivity (EC, μS/cm) using a water quality meter (AQUAREAD, AP‐800), and we used a chlorophyll meter (HL‐168C06, made in China) to measure the chlorophyll‐*a* (Chl‐a, μg/L).

The substrate samples of freshwater mussels were collected using a tubular shovel (total length: 47 cm; width of shovel: 11.5 cm; length of shovel: 15 cm). Then, the samples were emptied into bags and transported to the laboratory. In the laboratory, the substrate samples were first oven‐dried at 105°C for 24 hr (Gordon, Mcmahon, Finlayson, Gippel, & Nathan, 2004). Using three sizes of mesh sieves (4, 2, and 0.0625 mm), the substrate samples were sieved by handshaking for 30 min. According to Wentworth (1922), the substrate samples were divided into four groups: pebbles (>4 mm), granules (2–4 mm), sand (0.0625–2 mm), and silt (<0.0625 mm).

### 2.5 Data analyses

The sampling completeness of freshwater mussels for the study section was analyzed using abundance‐based rarefaction, as implemented in iNext Online (Chao, Ma, & Hsieh, 2016). Confidence intervals (95%) were calculated using 100 bootstrap replications.

Density of specimens in sampling area: *D* = *N*/*A*, biomass of specimens in sampling area: *B* = *W*/*A*, where *N*: the number of specimens in sampling area, *W*: the weight of specimens in sampling area, *A*: the sampling area (15 m^2^). Occurrence rates of species in all sampling sites: *O_i_* = *N_i_*/*S*; *N_i_*: the number of occurrence of *i* species in all sampling sites, *S*: all sampling sites (32). Extinction rates of species in all sampling sites: *E_i_* = *N_i_*/*S*; *N_i_*: the number of empty shells of *i* species in all sampling sites, *S*: the total number of *i* species in all sampling sites.

To analyze freshwater mussels diversity and richness in each sampled section, the relative abundance (*P_i_*), Shannon–Wiener index (*H′*), Simpson index (*D*), Margalef diversity index (*R*), and Pielou evenness index (*J′*) were calculated for each site (Magurran, 1988; Peet, 1974).

The beta diversity decomposition method was based on the Sørensen index (*β*_sor_), with its spatial turnover component (*β*_sim_) and nestedness component (*β*_sne_) (Baselga, 2010). The decomposition methods are shown as follows:

(1) $$\begin{array}{c} \beta_{\text{sor}}=\frac{b+c}{2a+b+c}\\ \beta_{\text{sim}}=\frac{\min\left(b,c\right)}{a+\min\left(b,c\right)}\\ \beta_{\text{sne}}=\frac{\left|b-c\right|}{2a+b+c}\times \frac{a}{a+\min\left(b,c\right)} \end{array}$$

where *a* is the number of common freshwater mussels among two study sections and *b* and *c* are the number of species present in only the a and b study sections, respectively.

Mantel tests (Legendre & Legendre, 2012) with 9,999 permutations (Spearman's method) were used to analyze the correlations of pairwise composition dissimilarity, spatial turnover, nestedness, density, biomass, and species number matrices and habitat characteristic matrices. R 3.2.0 (R Development Core Team, 2014) was used to perform all beta diversity analyses based on the BETAPART (Baselga & Orme, 2012) and VEGAN (Oksanen et al., 2015) packages.

One‐way analysis of variance (ANOVA) was used to detect differences in the density, biomass, and habitat characteristics of freshwater mussels in different areas based on SPSS. 22.0. Multidimensional scaling (MDS) was used to visualize changes in the assemblage structure of freshwater mussels based on PRIMER 6 (Clarke & Gorley, 2006).

Redundancy analysis (RDA) was used with 499 Monte Carlo permutations to evaluate variations in the assemblage composition, density, and biomass of freshwater mussels in relation to habitat characteristics based on CANOCO 4.5 (ter Braak & Verdonschot, 1995). All assemblage composition, density, and biomass of freshwater mussels and habitat characteristics were log10(*X* + 1)‐transformed to improve their normality before data analysis (ter Braak & Verdonschot, 1995).

## 3 RESULTS

### 3.1 Composition of freshwater mussel species

**Figure 2:**
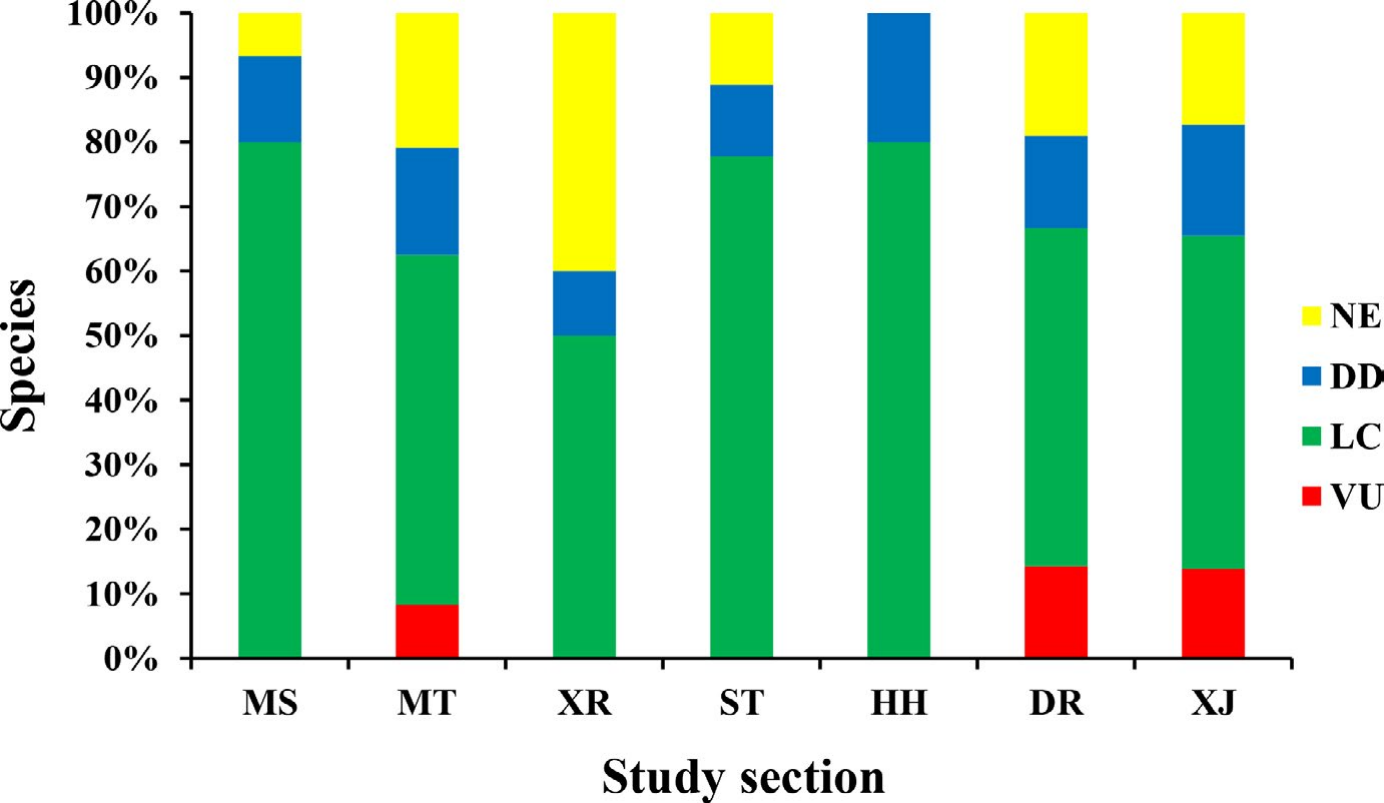
Percentage of freshwater mussels by IUCN category in the middle and lower reaches of the Xin River Basin. XJ: the middle and lower reaches of the Xin River Basin. Other river codes are the same as in Table 2

The number of freshwater mussels in the current period (2018; 29 species) was lower than it was in the historical period (1965–2013; 43 species; Tables 1 and S1). Unionidae was the most common family, accounting for 93.1% (27) in the total number of species (Table 1). Fourteen native species were not recorded in the current period (Table 1). Four freshwater mussel species (*Aculamprotula scripta* (Heude, 1875), *Aculamprotula tortuosa* (Lea, 1865), *Gibbosula polysticta* (Heude, 1877), and *Gibbosula rochechouartii* (Heude, 1875)) have been formally assessed using IUCN criteria, and their global status is currently listed as vulnerable species (IUCN, 2019; Table S1). These vulnerable species mainly presented in the MT and DR sections (Tables 1 and S1; Figure 2). Significant spatial changes were found among the number of freshwater mussel species in the current period (ANOVA, *F_df_*_1,_ *_df_*_2_ = 3.2, *p* = .022; Table S2). The MT had the highest number of native species, followed by the DR, and the number of native species in HH was the lowest (Table 2). The species accumulation curves for freshwater mussels in the study section were close to asymptotic based on relatively high sampling completeness and estimating Chao I as more than 95% of the study section (Figure S1).

**Table 1:** Composition, density, biomass, relative abundance, occurrence rate, and extinction rate of freshwater mussels in the middle and lower reaches of the Xin River Basin

Unionidae	Code	Density (ind/m^2^) (mean ± *SD*)	Biomass (g/m^2^) (mean ± *SD*)	Relative abundance (%)	Occurrence rate (%)	Extinction rate (%)
Unioninae						
*Aculamprotula* Wu et al., 1999						
*Aculamprotula scripta* (Heude, 1875)	AS	0	0	0	6.25	100.00
*Aculamprotula tortuosa* (Lea, 1865)	ATO	0	0	0	3.12	100.00
*Aculamprotula tientsinensis* (Crosse & Debeaux, 1863)	AT	0	0	0	3.12	100.00
*Acuticosta* Simpson, 1900						
*Acuticosta chinensis* (Lea, 1868)	AC	0.070 ± 0.121	0.650 ± 1.083	15.32	37.50	17.39
*Cuneopsis* Simpson, 1900						
*Cuneopsis celtiformis* (Heude, 1874)	CC	0	0	0	9.38	100.00
*Cuneopsis heudei* (Heude, 1874)	CH	0.007 ± 0.016	0.139 ± 0.341	2.42	15.62	66.67
*Cuneopsis pisciculus* (Heude, 1874)	CP	0.002 ± 0.004	0.039 ± 0.096	0.81	18.75	85.71
*Lepidodesma* Simpson, 1896						
*Lepidodesma languilati* (Heude, 1874)	LLA	0	0	0	6.25	100.00
*Nodularia* Conrad, 1853						
*Nodularia douglasiae* (Griffith & Pidgeon, 1833)	ND	0.069 ± 0.084	0.653 ± 0.884	21.77	75.00	41.30
*Schistodesmus* Simpson, 1900						
*Schistodesmus lampreyanus* (Baird & Adams, 1867)	SL	0.007 ± 0.012	0.206 ± 0.322	3.23	18.75	33.33
*Schistodesmus spinosus* (Simpson, 1900)	SS	0.002 ± 0.004	0.023 ± 0.056	0.81	3.12	0
Anodontinae						
*Anemina* Haas, 1969						
*Anemina arcaeformis* (Heude, 1877)	AA	0	0	0.81	31.25	90.91
*Anemina euscaphys* (Heude, 1879)	AE	0.004 ± 0.011	0.130 ± 0.318	2.42	6.25	40.00
*Anemina fluminea* (Heude, 1877)	AF	0	0	0	3.12	100.00
*Anemina globosula* (Heude, 1878)	AG	0.001 ± 0.004	0.078 ± 0.190	0.81	9.38	66.67
*Cristaria* Schumacher, 1817						
*Cristaria plicata* (Leach, 1815)	CP	0	0	0	56.25	100.00
*Lanceolaria* Conrad, 1853						
*Lanceolaria lanceolata* (Lea, 1856)	AL	0	0	0	15.62	100.00
*Lanceolaria gladiola* (Heude, 1877)	LGL	0	0	0	9.38	100.00
*Lanceolaria grayii* (Griffith & Pidgeon, 1833)	LG	0.008 ± 0.014	0.116 ± 0.181	1.61	37.50	83.33
*Lanceolaria triformis* (Heude, 1877)	LT	0	0	0	9.38	100.00
*Sinanodonta* Modell, 1945						
*Sinanodonta woodiana* (Lea, 1834)	SW	0.052 ± 0.055	1.333 ± 1.270	9.68	59.38	42.86
Gonideinae						
*Lamprotula* Simpson, 1900						
*Lamprotula caveata* (Heude, 1877)	LC	0.194 ± 0.308	7.553 ± 11.409	35.48	59.38	26.67
*Lamprotula leaii* (Griffith & Pidgeon, 1833)	LL	0.019 ± 0.046	0.406 ± 0.995	3.23	21.88	50.00
*Sinohyriopsis* Starobogatov, 1970						
*Sinohyriopsis cumingii* (Lea, 1852)	SC	0.007 ± 0.017	2.421 ± 5.930	1.61	31.25	84.62
*Solenaia* Conrad, 1869						
*Solenaia carinata* (Heude,1877)	SCA	0	0	0	9.38	100.00
*Solenaia oleivora* (Heude,1874)	SO	0	0	0	15.32	100.00
*Solenaia rivularis* (Heude,1877)	SR	0	0	0	9.38	100.00
Margaritiferidae						
*Gibbosula* Simpson, 1900						
*Gibbosula polysticta* (Heude, 1877)	AP	0	0	0	3.12	100.00
*Gibbosula rochechouartii* (Heude, 1875)	GR	0	0	0	9.38	100.00

**Table 2:** Density, biomass, relative abundance, and beta diversity pattern of freshwater mussels in the middle and lower reaches of the Xin River Basin

Sections	Code	Number of genera	Number of species	Number of native to China	Density (ind/m^2^) (mean ± *SD*)	Biomass (g/m^2^) (mean ± *SD*)	Relative abundance (%)	Beta diversity		
								*β*_sor_ (mean ± *SD*)	*β*_sim_ (mean ± *SD*)	*β*_sne_ (mean ± *SD*)
Main stem of the Xin River	MS	10	16	9	0.014 ± 0.041	0.140 ± 0.381	11.29	0.38 ± 0.09	0.14 ± 0.11	0.24 ± 0.15
Main tributary of the Xin River	MT	14	24	16	0.056 ± 0.153	1.841 ± 5.371	72.58	0.44 ± 0.16	0.08 ± 0.07	0.36 ± 0.22
Xi River	XR	7	10	3	0.008 ± 0.024	0.115 ± 0.364	5.65	0.37 ± 0.07	0.12 ± 0.13	0.25 ± 0.16
Santang River	ST	6	9	2	0.001 ± 0.004	0.024 ± 0.133	0.81	0.39 ± 0.13	0.13 ± 0.09	0.26 ± 0.14
Huhui River	HH	5	6	1	0.001 ± 0.003	0.056 ± 0.308	0.81	0.48 ± 0.16	0	0.48 ± 0.16
Dongda River	DR	12	22	14	0.009 ± 0.050	0.573 ± 3.138	8.87	0.40 ± 0.16	0.12 ± 0.08	0.28 ± 0.20
Total		14	29	21	0.015 ± 0.039	0.458 ± 1.432		0.61	0.24	0.38
Historical period		16	43	35	0.090 ± 0.080	2.531 ± 1.495				

### 3.2 Quantitative assessment of freshwater mussels

The occurrence rate of freshwater mussels was 87.5% in the study area. The occurrence rates of *Nodularia douglasiae* (Griffith & Pidgeon, 1833), *Lamprotula caveata* (Heude, 1877), *Sinanodonta woodiana* (Lea, 1834), and *Cristaria plicata* (Leach, 1815) were higher than they were for other mussels species, which indicated they were widespread species in this river (Table 1). The relative abundances of *Lamprotula caveata* (35.48%), *Nodularia douglasiae* (21.77%), and *Acuticosta chinensis* (Lea, 1868; 15.32%) were higher than they were for other mussel species, which indicated they were dominant species in the study area (Table 1). However, 15 freshwater mussels were empty shells, and the extinction rates of only seven freshwater mussels were lower than 50% (Table 1).

Significant differences were found among the density of 29 freshwater mussels (ANOVA, *F_df_*_1,_ *_df_*_2_ = 3.832, *p* = .010; Table S2). The mean density in *Lamprotula caveata* was the highest (0.194 ind./m^2^), followed by *Acuticosta chinensis* (0.070 ind./m^2^) and *Nodularia douglasiae* (0.069 ind./m^2^) in the study section (Table 1). The mean biomass in *Lamprotula caveata* (7.553 g/m^2^), *Sinohyriopsis cumingii* (Lea, 1852; 2.421 g/m^2^), and *Sinanodonta woodiana* (1.333 g/m^2^) was higher than it was in other freshwater mussels (Table 1).

The total density and biomass of freshwater mussels in the current period were 0.015 ind./m^2^ and 0.458 mg/L in the study area, respectively, which were lower than they were in the historical period (0.090 ind./m^2^ and 2.531 mg/L, respectively; Table 2). Significant spatial changes were found among the density of freshwater mussels in the current period (ANOVA, *F_df_*_1,_ *_df_*_2_ = 3.832, *p* = .010; Table S2). The MT had the greatest density and biomass, followed by the MS, and the density and biomass in the HH and ST were the lowest (Table 2).

### 3.3 Diversity of freshwater mussels

**Figure 3:**
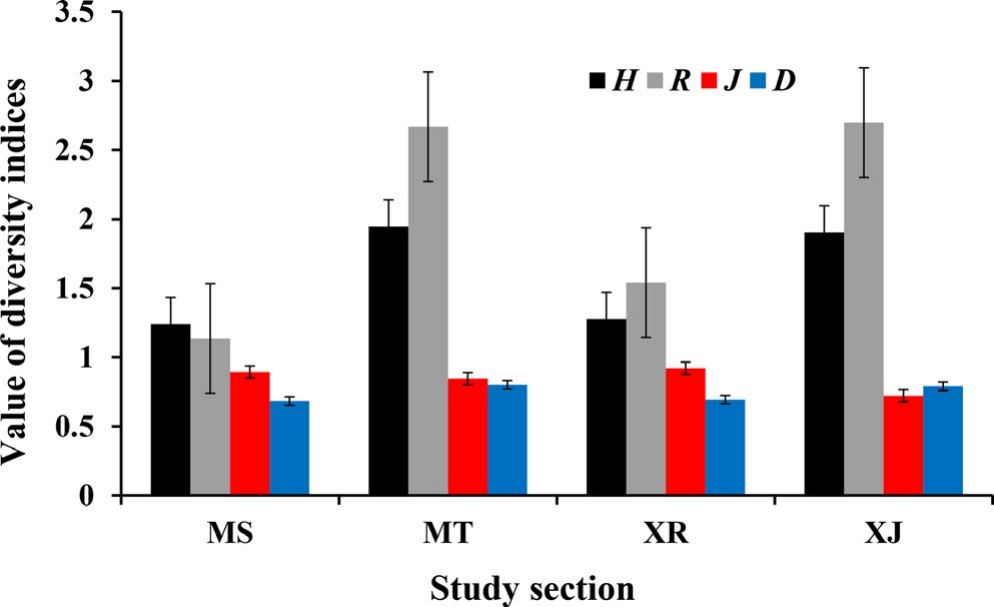
Spatial change in the diversity of freshwater mussels in the middle and lower reaches of the Xin River Basin. Diversity indices were not included ST, HH, DD, because they were not enough data. XJ: the middle and lower reaches of the Xin River Basin. Other river codes are the same as in Table 2

The diversity and abundance in the MT were higher than they were in other sections (Figure 3). The freshwater mussel composition dissimilarity had a total value of 0.61 (Table 2). The composition dissimilarity in the MT and HH (0.44 and 0.48) was higher than they were in other sections (Table 2). The nestedness component was greater than the spatial turnover component in each section (Table 2).

### 3.4 Association of the assemblage structure of freshwater mussels and habitat characteristics

**Figure 4:**
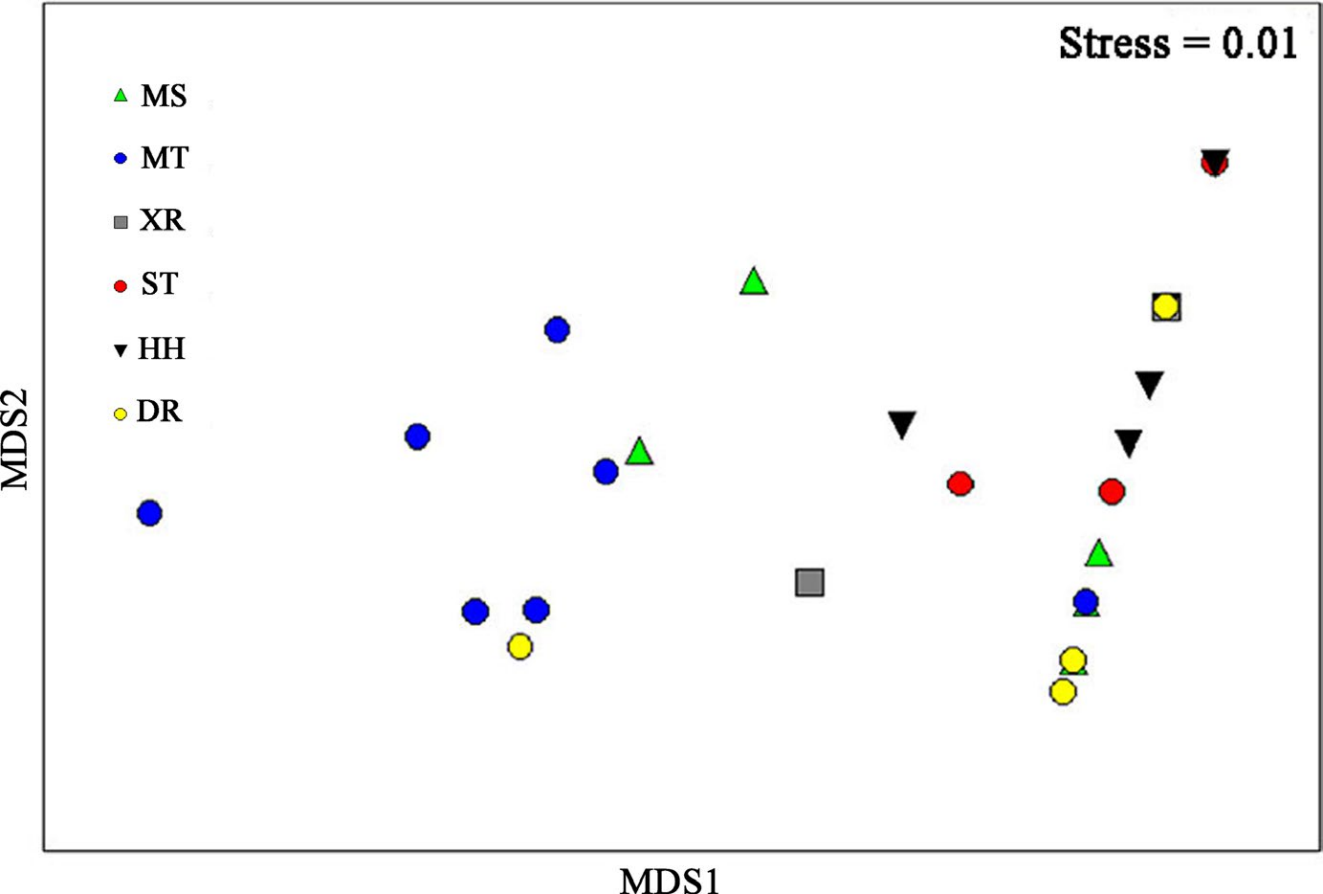
Metric multidimensional scaling (MDS) ordination of the freshwater mussel assemblage structure in the middle and lower reaches of the Xin River Basin. River codes are the same as in Table 2

**Figure 5:**
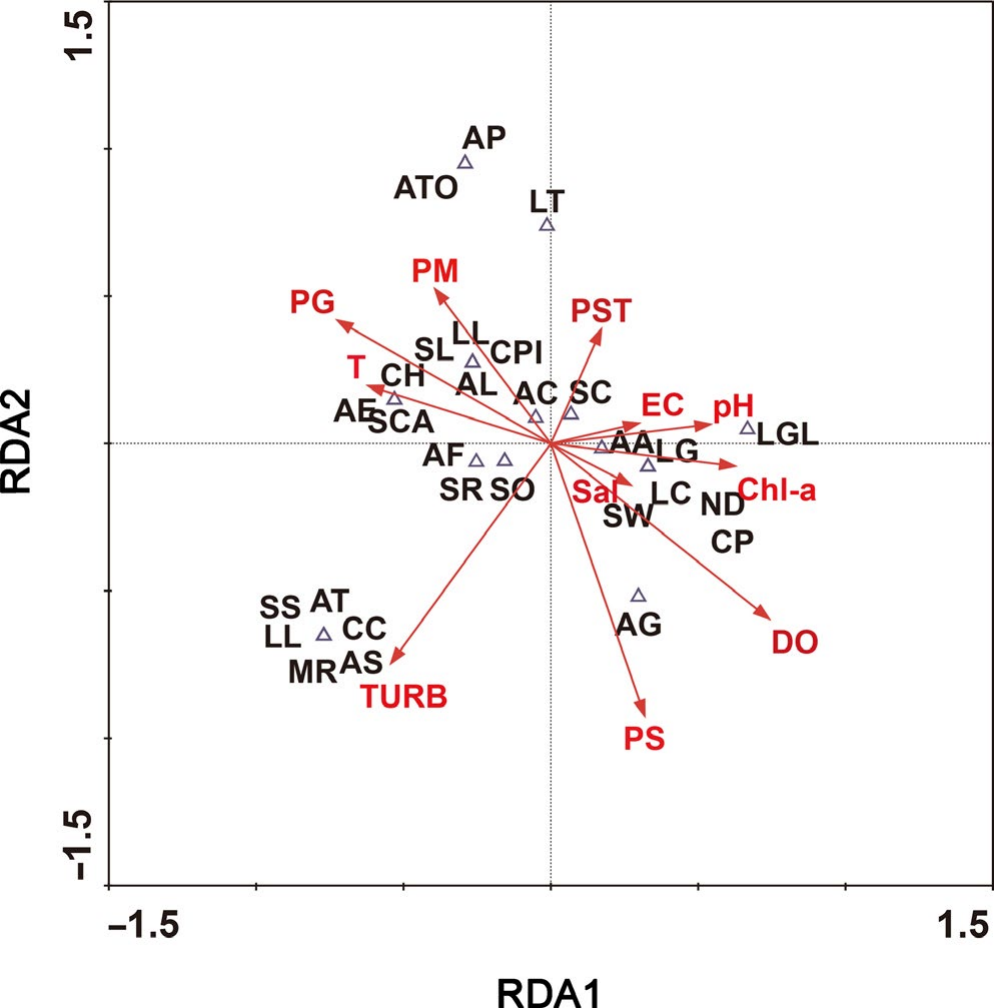
Ordination biplot of assemblage structure of freshwater mussels and habitat characteristics obtained by RDA across sampling sites in the middle and lower reaches of the Xin River Basin. Habitat characteristic codes are the same as in Table 3. Species codes are the same as in Table 1

The assemblage structure of freshwater mussels formed two groups, MS and MT, and the other group formed the second cluster (Figure 4). Significant differences were detected in the turbidity (ANOVA, *F_df_*_1,_ *_df_*_2_ = 2.300, *p* = .038), water temperature (ANOVA, *F_df_*_1,_ *_df_*_2_ = 3.670, *p* = .012), salinity (ANOVA, *F_df_*_1,_ *_df_*_2_ = 13.176, *p* < .001), electrical conductivity(ANOVA, *F_df_*_1,_ *_df_*_2_ = 12.667, *p* < .001), pH (ANOVA, *F_df_*_1,_ *_df_*_2_ = 6.534, *p* < .001), and stone (ANOVA, *F_df_*_1,_ *_df_*_2_ = 3.156, *p* = .023) in the study section (Tables 3 and S2). Redundancy analysis (RDA) showed that the assemblage structure of freshwater mussels was correlated with habitat characteristics (Figure 5). Nine freshwater mussels were correlated with turbidity, and 12 freshwater mussels were correlated with substrate characteristics (Figure 5). Eight freshwater mussels were correlated with physicochemical parameters (Figure 5). In addition, dissolved oxygen was significantly associated with the species number and beta diversity pattern, and PM was significantly associated with the density and relative abundance of freshwater mussels, based on the Mantel test (*p* < .05; Table 4).

**Table 3:** Mean ± *SD* values of physicochemical parameter and substrate characteristics of freshwater mussels in different areas of the middle and lower reaches of the Xin River Basin

Variables	Code	MS	MT	XR	ST	HH	DR
Turbidity	TURB (NTU^+^)	9.86 ± 3.79	10.68 ± 20.77	9.15 ± 8.56	3.14 ± 1.11	6.02 ± 3.60	3.35 ± 1.37
Water temperature	*T* (℃)	22.27 ± 2.11	25.30 ± 1.11	19.70 ± 4.03	24.88 ± 2.19	21.36 ± 4.27	25.91 ± 2.31
Salinity	Sal (mg/L)	0.05 ± 0.01	0.05 ± 0.01	0.06 ± 0.01	0.04 ± 0.01	0.08 ± 0.01	0.05 ± 0.01
Dissolved oxygen	DO (mg/L)	8.20 ± 0.70	8.19 ± 0.54	8.46 ± 2.04	8.97 ± 0.83	9.34 ± 1.15	7.77 ± 0.48
Electrical conductivity	EC (μS/cm)	170.29 ± 21.88	148.00 ± 14.36	192.00 ± 36.77	114.80 ± 21.55	233.67 ± 46.54	157.25 ± 4.65
Chlorophyll‐*a*	Chl‐*a* (μg/L)	11.72 ± 19.86	18.05 ± 26.86	13.43 ± 14.16	97.82 ± 138.01	102.38 ± 127.44	37.55 ± 53.90
Hydrogen ion concentration	pH	7.56 ± 0.31	6.97 ± 0.18	6.96 ± 0.04	7.20 ± 0.14	7.47 ± 0.43	6.87 ± 0.09
% of mud	PM	0.062 ± 0.063	0.166 ± 0.181	0.057 ± 0.048	0.198 ± 0.264	0.059 ± 0.065	0.346 ± 0.232
% of sand	PS	0.440 ± 0.214	0.608 ± 0.240	0.664 ± 0.257	0.733 ± 0.288	0.613 ± 0.246	0.378 ± 0.117
% of gravel	PG	0.051 ± 0.034	0.080 ± 0.063	0.028 ± 0.003	0.037 ± 0.044	0.068 ± 0.044	0.100 ± 0.034
% of stone	PST	0.447 ± 0.284	0.145 ± 0.140	0.251 ± 0.302	0.031 ± 0.059	0.260 ± 0.217	0.176 ± 0.134

Note

River codes are the same as in Table 2.

**Table 4:** Effects of physicochemical parameter and substrate characteristics on pairwise species number, density, biomass, relative abundance, compositional dissimilarity, spatial turnover, and nestedness components obtained from BAS frameworks in the middle and lower reaches of the Xin River Basin, China

Variables	*N*	*B*	*D*	*P_i_*	*β*_sor_	*β*_sim_	*β*_sne_
*T*							
*r*	.08	−.03	−.20	−.16	−.04	−.16	.06
*p*	.26	.59	.15	.30	.50	.24	.37
TURB							
*r*	−.03	.16	.32	.29	−.11	.03	−.10
*p*	.59	.35	.12	.21	.41	.42	.39
Sal							
*r*	.19	−.32	−.28	−.28	.38	−.58	.57
*p*	.29	.20	.39	.44	.06	.15	.09
DO							
*r*	.51*	−.15	−.18	−.20	.69**	−.36	.68**
*p*	**.03**	.49	.46	.49	**.01**	.15	**.01**
EC							
*r*	.13	−.25	−.24	−.23	.24	−.49	.44
*p*	.33	.42	.44	.47	.15	.10	.10
Chl‐*a*							
*r*	.21	−.16	−.04	−.10	.37	−.22	.38
*p*	.16	.37	.53	.62	.14	.14	.10
pH							
*r*	−.03	−.13	−.22	−.21	.10	.17	−.02
*p*	.61	.42	.17	.20	.25	.24	.50
PM							
*r*	.01	−.04	−.30**	−.22*	−.15	−.02	−.10
*p*	.45	.77	**.01**	**.03**	.41	.34	.54
PS							
*r*	−.07	−.27	−.31	−.31	.05	.41	−.18
*p*	.42	.31	.31	.26	.35	.09	.30
PG							
*r*	.22	.09	−.17	−.12	.05	−.01	.04
*p*	.15	.38	.34	.37	.33	.49	.40
PST							
*r*	−.31	−.23	−.21	−.22	−.30	.27	.35
*p*	.19	.49	.58	.59	.09	.25	.16

Note

Significant results are in bold (*p < .05; **p < .01). *N*, number of species; *B*, biomass; *D*: density; *P_i_*, relative abundance; *β*_sor_, compositional dissimilarity; *β*_sim_, spatial turnover component; *β*_sne_, nestedness component. Habitat characteristic codes are the same as in Table 3.

## 4 DISCUSSION

### 4.1 Changes in the diversity of freshwater mussels

Poyang Lake is a biodiversity hotspot for mollusks in East Asia with at least 155 species known, of which more than 50% were native species of bivalve and gastropod (Lin, 1962; Liu et al., 1979; Tchang & Li, 1965; Wu et al., 1994; Xiong et al., 2013; Xu, 2013). The Xin River Basin has at least 72 known mollusk species, including 24 gastropod species and 48 bivalve species (Zhang et al., 2013). However, due to disturbance from natural and human factors, populations of many species have been extirpated or are rapidly decreasing in these areas (Shu et al., 2009; Wu, Liang, Wang, Xie, & Ouyang, 2000; Xiong et al., 2013). In this study, compared to the historical period, the number of species, density, and biomass of freshwater mussels in the current period decreased 32.6%, 83%, and 82%, respectively (Tchang & Li, 1965; Wu et al., 1994; Xiong et al., 2013; Xu, 2013; Zhang et al., 2013). These results indicated that populations of freshwater mussel species have been declining in the current period. The declining freshwater mussel diversity may be attributed to dam constructions, sand dredging, land use, and water pollution (Xiong et al., 2013; Zhang et al., 2013). Dam constructions and sand dredging could alter river morphology and destabilize substrates, resulting in declines in some freshwater mussels (Downward & Skinner, 2005; Hartfield, 2010), and also may block host fish from distributing mussels (Lydeard et al., 2004; Williams, Bogan, & Garner, 2008). Moreover, land use in different regions differs in intensity. Libois and Hallet‐Libois (1987) revealed that the high proportion of agriculture and urbanization in the lower reaches in Belgium will lead to a decrease in the freshwater mussel population.

### 4.2 Key factors for determining spatial heterogeneity of freshwater mussel community

Habitat characteristics are important for determining freshwater mussel assemblage structure (Vaughn, 2018). The spatial difference in the ecological environment and the complexity of the habitat determine the assemblage structure of freshwater mussels (Haag, 2012; Vaughn, 2012). This study revealed that the assemblage structure of freshwater mussels in the study section showed spatial differences. Many studies showed that the diversity of bivalves in the lower section was higher than it was in the upper and middle sections of the river (Daniel & Brown, 2014; Rahel & Hubert, 1991). For example, Xiao et al. (2013) report that the number of bivalve species in the Ganjiang River increases along the upper reaches of the river to the estuary. Similarly, this study also showed a consistent pattern, which may be attributed to complex habitat heterogeneity in the lower area of the Xin River Basin.

The dispersal activity of freshwater mussels was relatively weak (Vaughn, 2012). Most of them are sensitive to the environment, and environmental change affects their assemblage composition and distribution. Species with stronger adaptability to environmental change may become dominant in this area (Bogan, 2008; Bogan & Roe, 2016). Some studies have also shown that habitat characteristics, such as substrate and environmental factors, have significantly affected the distribution of freshwater mussels (Akiyama & Maruyama, 2010; Campbell & Prestegaard, 2016; Martin, Larry, & Björnl, 2008; Nakano, Takakura, Morii, & Urabe, 2017; Negi & Mamgain, 2013; Vaughn, 2018). For example, Akiyama and Maruyama (2010) and Xiong et al. (2013) revealed that freshwater mussels likely occur in muddy areas with abundant organic matter. Österling, Martin, and Arvidsson (2008) revealed that the number of young individuals of freshwater mussels in Sweden was more obvious in the waters with high turbidity than the low turbidity. Sheldon and Walker (1989) compared two freshwater mussels in Australia and found they respond metabolically to low DO very differently from each other. This study also showed that the distribution of freshwater mussels was correlated with environmental factors. Moreover, some studies showed the change of hydrological dynamics scoured the substrates of the mussels in the United States, resulting in a decrease in the number of mussel species in the river (Strayer & Ralley, 1993). The proportion of the substrate composition can also effectively predict the distribution of mussels in rivers (Hastie & Young, 2003). Generally, mussels like habitats in the substrates where the sediment is particularly stable in Scotland or the United Kingdom (Morales, Weber, Mynett, & Newton, 2006; Strayer, 1999). The distribution of freshwater mussels in shallow water areas with high percentage of mud and silt bottoms may be relatively concentrated in the United States (Strayer, 2008). This study also showed that the number of species in the MT and DR with relatively high percentage of mud and silt bottoms was higher than in other sections.

### 4.3 Threat factors of freshwater mussel diversity

The main threats to freshwater mussels are human activities, including habitat loss and fragmentation, overfishing, water pollution and eutrophication, invasive species, climate change, and overfishing (Dudgeon et al., 2006; Galbraith, Spooner, & Vaughn, 2010; Lopes‐Lima et al., 2017; Modesto et al., 2018; Zieritz et al., 2016). In addition, barriers between rivers and lakes, and the loss of host fish are important factors leading to the decline in mussel species (Lopes‐Lima et al., 2017). Dam construction has caused habitat fragmentation and loss and has been shown to have a profound effect on the survival of freshwater mussels (Haag, 2012). The Jiepai Dam was constructed in the Xin River Basin, which significantly changed hydrological conditions in the middle area, affecting the assemblage structure of freshwater mussels (Zou, Tang, & Chen, 2018). Many studies have shown that sand dredging changed aquatic habitats and decreased species richness by 30%–70% and the abundance and biomass of macrozoobenthos by 40%–95% (Johnson, Jin, Carreiro, & Jack, 2012; Li et al., 2019; Narin & Michel, 2009). Many sand‐dredging boats focused their extractions in the study area (Li et al., 2019), which changed substrate conditions and directly affected the assemblage structure of freshwater mussels (Xiong et al., 2013; Zhang et al., 2013). Moreover, due to the acceleration of urbanization and agriculture, industrial wastewater and domestic sewage have resulted in habitat deterioration and eutrophication, indirectly affecting the assemblage structure of freshwater mussels (Hu, Zhou, Wang, & Wei, 2010; Li et al., 2019; Wan & Jiang, 2005). Freshwater mussels have great economic value, such as providing a direct source of protein, and valuable materials (shells and pearls); however, overharvest has greatly damaged mussel resources (Bogan, 2008; Vaughn, 2018). *Cristaria* and *Sinohyriopsis* species were used to make pearls and provide food resources; *Lamprotula*, *Aculamprotula*, *Gibbosula*, *Cuneopsis*, and *Lanceolaria* species were used to make buttons; *Solenaia* species were used to provide food resources; and many small mussels have been discarded in random piles on the shore (Xiong et al., 2013; Zhang et al., 2013). Fish diversity plays an important role in determining freshwater mussel diversity because of the mussels' parasitic life cycle (Cao et al., 2018; Lopes‐Lima et al., 2017; Vaughn, Atkinson, & Julian, 2015). The harvesting of host fish laden with encysted glochidia likely has detrimental effects on the reproduction, distribution, and dispersal of freshwater mussels across the region (Audzijonyte, Kuparinen, Gorton, & Fulton, 2013; Blažek & Gelnar, 2006). In this study, 14 native freshwater mussels were not found and 52% of freshwater mussel species were empty shells. At the same time, *Aculamprotula scripta*, *Aculamprotula tortuosa*, *Gibbosula polysticta*, and *Gibbosula rochechouartii* are currently listed as vulnerable species using IUCN criteria.

### 4.4 Conservation and management implications

Freshwater mussels are considered of the most threatened freshwater organisms globally (Bogan, 2008; Zieritz et al., 2018). To date, freshwater mussels have rarely been conserved in East and South‐East Asia (Cao et al., 2018; IUCN, 2019; Zieritz et al., 2018). Only four countries in these regions have them on red lists (Vietnam, Korea, Japan, and Russia), and 228 species are not under international legal protection (Zieritz et al., 2018).

Given the declining freshwater mussel diversity, we suggest its biodiversity conservation should be carried out in the following ways: (a) establishing a nature reserve. Habitat loss and fragmentation are the most important factors leading to the species extinction of freshwater mussels (Dudgeon et al., 2006; Jones & Neves, 2011; Lopes‐Lima et al., 2017; Vaughn, 2012). Habitats with abundant endemic species should be identified as nature reserves. The MT should be established as one large protected area because the nestedness component was the main pattern of beta diversity in this study. (b) Habitat protection. To reduce the effect of human activity on the habitat, sand mining should be regulated and managed, and the natural hydrological rhythm should be maintained to keep the balance (Li et al., 2019). (c) Because sampling conditions are difficult in the Xin River Basin, novel molecular tools such as environmental DNA (Jerde, Mahon, Chadderton, & Lodge, 2011) might be crucial for detecting rare species; (d) Reproductive biology research. Reproductive biology is very important for the conservation of freshwater mussels (Vaughn, 2012). One important limiting factor of protection work is the lack of information about which mussels use host fish and the ease of artificial propagation and release.

## CONFLICT OF INTEREST

None declared.

## AUTHOR CONTRIBUTIONS

WS, XL, SO, and XW conceived the study. All authors contributed to the study design and data collection. WS and XL analyzed the data. WS, XL, SO, and XW led the writing of the manuscript.

## Data Availability

The data used in this study are archived in the Dryad Data Repository (https://doi.org/10.5061/dryad.47d7wm38g).
