# Supplementary figures and images for: Declining freshwater mussel diversity in the middle and lower reaches of the Xin River Basin: Threat and conservation
Source: Ecol Evol. 2019 Nov 21;9(24):14142–53. doi: 10.1002/ece3.5849 (PMC6953653; doi:10.1002/ece3.5849)

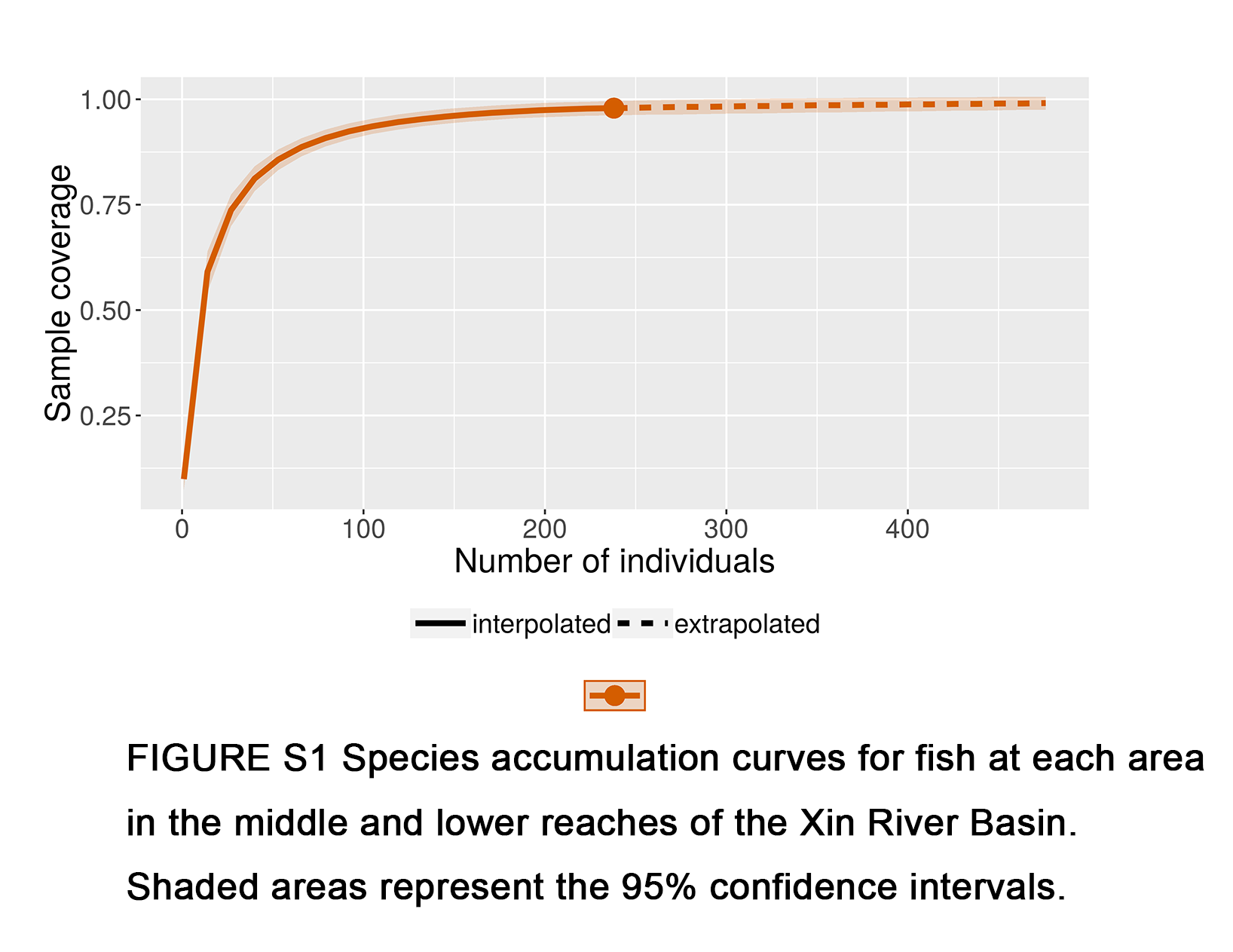

Supplement: Supplementary file 1 [file ECE3-9-14142-s001.tif]
