# Supplementary material for: Declining freshwater mussel diversity in the middle and lower reaches of the Xin River Basin: Threat and conservation
Source: Ecol Evol. 2019 Nov 21;9(24):14142–53. doi: 10.1002/ece3.5849 (PMC6953653; doi:10.1002/ece3.5849)
Supplement: Supplementary file 2 [file ECE3-9-14142-s002.docx]

**TABLE S1** Presence or absence, native to China and IUCN Global conservation status of freshwater mussels in the historical (1965-2013) and current periods (2018) from the middle and lower reaches of the Xin River Basin. River codes were the same as in Table 2. DD: Data Deficient; LC: Least Concern; CR: Critically Endangered; VU: Vulnerable; NE: No evaluation. Native to China: references from Liu et al. (1979), Cai & Huang 1991, Hu (2005), Graf & Cummings (2018).

|  | Current period (2018) | | | | | | Historical period (1965-2013) | Native to China | IUCN Global conservation status |
| --- | --- | --- | --- | --- | --- | --- | --- | --- | --- |
|  | MS | MT | XR | ST | HH | DR |  |  |  |
| **Unionidae** |  |  |  |  |  |  |  |  |  |
| ***Nodularia*** **Conrad, 1853** |  |  |  |  |  |  |  |  |  |
| *Nodularia douglasiae* (Griffith & Pidgeon, 1833) | + | + | + | + | + | + | + |  | LC |
| ***Lanceolaria*** **Conrad, 1853** |  |  |  |  |  |  |  |  |  |
| *Lanceolaria grayii* (Griffith & Pidgeon, 1833) | + | + | + | + | + | + | + |  | LC |
| *Lanceolaria triformis* (Heude, 1877) | + |  |  |  |  | + | + | √ | DD |
| *Lanceolaria gladiola* (Heude, 1877) | + |  |  | + |  |  | + |  | LC |
| *Lanceolaria lanceolata* (Lea, 1856) | + | + |  |  |  | + | + | √ | LC |
| *Lanceolaria eucylindrica* Lin, 1962 |  |  |  |  |  |  | + | √ | NE |
| ***Acuticosta*** **Simpson, 1900** |  |  |  |  |  |  |  |  |  |
| *Acuticosta chinensis* (Lea, 1868) | + | + | + |  |  | + | + | √ | NE |
| *Acuticosta trisulcata* (Heude,1883) |  |  |  |  |  |  | + | √ | NE |
| ***Schistodesmus*** **Simpson, 1900** |  |  |  |  |  |  |  |  |  |
| *Schistodesmus lampreyanus* (Baird & Adams, 1867) | + | + |  |  |  | + | + | √ | LC |
| *Schistodesmus spinosus* (Simpson, 1900) |  | + |  |  |  |  | + | √ | LC |
| ***Cristaria*** **Schumacher, 1817** |  |  |  |  |  |  |  |  |  |
| *Cristaria plicata* (Leach, 1815) | + | + | + | + | + | + | + |  | DD |
| ***Sinanodonta*** **Modell, 1945** |  |  |  |  |  |  |  |  |  |
| *Sinanodonta woodiana* (Lea, 1834) | + | + | + | + | + | + | + |  | NE |
| *Sinanodonta lucida* (Heude, 1887) |  |  |  |  |  |  | + | √ | NE |
| ***Anemina*** **Haas, 1969** |  |  |  |  |  |  |  |  |  |
| *Anemina globosula* (Heude, 1878) |  | + | + | + |  |  | + | √ | NE |
| *Anemina fluminea* (Heude, 1877) | + |  |  |  |  |  | + | √ | LC |
| *Anemina euscaphys* (Heude, 1879) |  | + |  |  |  | + | + |  | DD |
| *Anemina arcaeformis* (Heude,1877) | + | + | + | + |  | + | + |  | LC |
| ***Cuneopsis* Simpson, 1900** |  |  |  |  |  |  |  |  |  |
| *Cuneopsis heudei* (Heude,1874) |  | + |  |  |  | + | + | √ | LC |
| *Cuneopsis pisciculus* (Heude, 1874) | + | + |  |  |  | + | + | √ | LC |
| *Cuneopsis celtiformis* (Heude, 1874) |  | + |  |  |  |  | + | √ | LC |
| *Cuneopsis rufescens*(Heude, 1874) |  |  |  |  |  |  | + | √ | VU |
| *Cuneopsis capitatus*(Heude,1874) |  |  |  |  |  |  | + | √ | LC |
| *Cuneopsis kiangsiensis* Tchang&Li,1965 |  |  |  |  |  |  | + | √ | NE |
| ***Sinohyriopsis* Starobogatov, 1970** |  |  |  |  |  |  |  |  |  |
| *Sinohyriopsis cumingii* (Lea, 1852) | + | + |  | + |  | + |  | √ | LC |
| ***Lamprotula*** **Simpson, 1900** |  |  |  |  |  |  |  |  |  |
| *Lamprotula caveata* (Heude, 1877) | + | + | + | + | + | + | + | √ | LC |
| *Lamprotula leaii* (Griffith & Pidgeon, 1833) | + | + |  |  |  | + | + | √ | LC |
| *Lamprotula microsticta*(Heude, 1877) |  |  |  |  |  |  | + | √ | NE |
| *Lamprotula triclava* (Heude, 1877) |  |  |  |  |  |  | + | √ | CR |
| *Lamprotula bazini* (Heude,1877) |  |  |  |  |  |  | + | √ | DD |
| ***Aculamprotula*** **Wu et al., 1999** |  |  |  |  |  |  |  |  |  |
| *Aculamprotula tientsinensis* (Crosse & Debeaux, 1863) |  | + |  |  |  |  | + | √ | DD |
| *Aculamprotula scripta* (Heude, 1875) |  | + |  |  |  |  | + | √ | VU |
| *Aculamprotula tortuosa* (Lea, 1865) |  |  |  |  |  | + | + | √ | VU |
| *Aculamprotula fibrosa*(Heude,1877) |  |  |  |  |  |  | + | √ | LC |
| *Aculamprotula zonata*(Heude, 1883) |  |  |  |  |  |  | + | √ | DD |
| ***Solenaia*** **Conrad, 1869** |  |  |  |  |  |  |  |  |  |
| *Solenaia carinata* (Heude,1877) |  | + |  |  |  | + | + | √ | NE |
| *Solenaia oleivora* (Heude,1874) |  | + | + |  |  | + | + |  | NE |
| *Solenaia rivularis* (Heude,1877) |  | + | + |  |  | + | + | √ | NE |
| *Solenaia triangularis* (Heude,1885) |  |  |  |  |  |  | + |  | NE |
| ***Lepidodesma*** **Simpson, 1896** |  |  |  |  |  |  |  |  |  |
| *Lepidodesma languilati* (Heude, 1874) |  | + |  |  |  |  |  | √ | DD |
| ***Ptychorhynchus*** **Simpson, 1900** |  |  |  |  |  |  |  |  |  |
| *Ptychorhynchus pfisteri* (Heude,1874) |  |  |  |  |  |  | + | √ | NE |
| ***Pseudodon* Gould, 1844** |  |  |  |  |  |  |  |  |  |
| *Pseudodon secundus* (Heude, 1877) |  |  |  |  |  |  | + | √ | NE |
| **Margaritiferidae** |  |  |  |  |  |  |  |  |  |
| ***Gibbosula* Simpson, 1900** |  |  |  |  |  |  |  |  |  |
| *Gibbosula rochechouartii* (Heude, 1875) |  | + |  |  |  |  | + | √ | VU |
| *Gibbosula polysticta* (Heude, 1877) |  |  |  |  |  | + | + | √ | VU |
