# Supplementary material for: Declining freshwater mussel diversity in the middle and lower reaches of the Xin River Basin: Threat and conservation
Source: Ecol Evol. 2019 Nov 21;9(24):14142–53. doi: 10.1002/ece3.5849 (PMC6953653; doi:10.1002/ece3.5849)
Supplement: Supplementary file 3 [file ECE3-9-14142-s003.docx]

**TABLE S2** The one-way analysis of variance (ANOVA) analysis of number of species, biomass, density, physicochemical parameters and substrate characteristic of freshwater mussels in the middle and lower reaches of the Xin River Basin. Significant results are in bold (**P* < 0.05; ***P* < 0.01, ****P*< 0.001).

|  | df1(sections) | df2(residuals) | *F* | *P* |
| --- | --- | --- | --- | --- |
| Number of species | 5 | 27 | 3.187 | **0.022*** |
| Biomass | 5 | 27 | 1.876 | 0.133 |
| Density | 5 | 27 | 3.832 | **0.010**** |
| Turbidity | 5 | 27 | 2.300 | **0.038*** |
| Water temperature | 5 | 27 | 3.670 | **0.012*** |
| Salinity | 5 | 27 | 13.176 | **＜0.001***** |
| Dissolved oxygen | 5 | 27 | 2.408 | 0.064 |
| Electrical conductivity | 5 | 27 | 12.667 | **＜0.001***** |
| Chlorophyll-a | 5 | 27 | 1.477 | 0.231 |
| pH | 5 | 27 | 6.534 | **＜0.001***** |
| % of mud | 5 | 27 | 2.291 | 0.074 |
| % of sand | 5 | 27 | 1.792 | 0.148 |
| % of gravel | 5 | 27 | 1.542 | 0.210 |
| % of stone | 5 | 27 | 3.156 | **0.023*** |
